# Sleep Promotes Consolidation of Emotional Memory in Healthy Children but Not in Children with Attention-Deficit Hyperactivity Disorder

**DOI:** 10.1371/journal.pone.0065098

**Published:** 2013-05-29

**Authors:** Alexander Prehn-Kristensen, Manuel Munz, Ina Molzow, Ines Wilhelm, Christian D. Wiesner, Lioba Baving

**Affiliations:** 1 Department of Child and Adolescent Psychiatry and Psychotherapy, Center for Integrative Psychiatry, School of Medicine, Christian-Albrechts-University Kiel, Germany; 2 Institute of Medical Psychology and Behavioural Neurobiology, University of Tübingen, Germany; 3 Child Development Center, University Children's Hospital Zürich, Switzerland; University of Pennsylvania, United States of America

## Abstract

Fronto-limbic brain activity during sleep is believed to support the consolidation of emotional memories in healthy adults. Attention deficit-hyperactivity disorder (ADHD) is accompanied by emotional deficits coincidently caused by dysfunctional interplay of fronto-limbic circuits. This study aimed to examine the role of sleep in the consolidation of emotional memory in ADHD in the context of healthy development. 16 children with ADHD, 16 healthy children, and 20 healthy adults participated in this study. Participants completed an emotional picture recognition paradigm in sleep and wake control conditions. Each condition had an immediate (baseline) and delayed (target) retrieval session. The emotional memory bias was baseline–corrected, and groups were compared in terms of sleep-dependent memory consolidation (sleep vs. wake). We observed an increased sleep-dependent emotional memory bias in healthy children compared to children with ADHD and healthy adults. Frontal oscillatory EEG activity (slow oscillations, theta) during sleep correlated negatively with emotional memory performance in children with ADHD. When combining data of healthy children and adults, correlation coefficients were positive and differed from those in children with ADHD. Since children displayed a higher frontal EEG activity than adults these data indicate a decline in sleep-related consolidation of emotional memory in healthy development. In addition, it is suggested that deficits in sleep-related selection between emotional and non-emotional memories in ADHD exacerbate emotional problems during daytime as they are often reported in ADHD.

## Introduction

Attention deficit hyperactivity disorder (ADHD) is one of the most frequently diagnosed psychiatric disorders in children and adolescents. ADHD is characterized by symptoms of inattention, hyperactivity, and/or impulsiveness [1] and it is often accompanied by emotional problems [2]–[4]. Imaging studies in ADHD revealed not only alterations in the structure and function of the prefrontal cortex, but also in the amygdala and the hippocampus [5]–[8] which are both critically involved in emotional processes [9]–[11]. In particular, the affected interplay between these regions is assumed to cause emotional problems in ADHD during daytime [12]. Moreover, ADHD is associated with sleep disorders [13], [14], and has to be seen as a “24h syndrome” which also affects sleep and its functions [15], [16].

Sleep facilitates the consolidation of emotional memory in healthy children and adults [17]–[19]. Better performance in emotional memory is coincided by an integration of PFC, amygdala, and the hippocampus during sleep [20]. Sleep electro-encephalogram (EEG) studies revealed that hippocampus-related memory consolidation is supported by slow frontal oscillation activity (<1Hz) during slow wave sleep (SWS) [21], [22], while amygdala-associated memory consolidation is supported by frontal theta oscillations during REM sleep [23]–[25].

Sleep and its memory-related functions underlie ontogenetic changes: SWS and REM sleep occur less and less frequently from infancy to adulthood [26], and it is proposed that children benefit more from sleep than adults do with respect to hippocampus-dependent memory due to increased SWS [27]. In accordance with changes in sleep-stage duration, oscillatory slow waves, as well as theta activity during sleep, decrease from childhood to adulthood [28]–[30]. While children benefit from sleep with respect to emotional memory [19], it is still unclear whether healthy children benefit more from sleep with respect to emotional memory consolidation than healthy adults do. ADHD, in turn, is not only characterised by deviant activity in frontal brain circuits during the daytime [31], [32] but also by imbalanced anterior-posterior slow wave activity during sleep [33]. As shown recently, young patients suffering from ADHD displayed deficits in the sleep-dependent consolidation of declarative memory which was explained by dysfunctional support of oscillatory EEG activity on sleep-dependent memory consolidation [16]. If frontal oscillatory brain activity does indeed drive the consolidation of emotional memories during sleep, then children with ADHD should display deficits in sleep-related emotional memory consolidation.

The present study was designed to investigate the sleep-associated consolidation of emotional memory during sleep in children with ADHD in the context of healthy development. We hypothesize that healthy children particularly benefit from sleep due to enhanced frontal EEG activity (slow oscillation power during SWS and theta activity during REM sleep). Furthermore, we presume that sleep in children with ADHD does not benefit the consolidation of emotional memory. At the same time, we expect that frontal oscillatory activity during sleep does not support memory performance in children with ADHD as it is assumed to do in healthy individuals. A lack of sleep-dependent consolidation of emotional memory would offer new insights into aberrant emotional development in children with ADHD.

## Methods

### Ethics Statement

All participants or, in the case of participating children, their parents gave written informed consent, and participants were reimbursed with a voucher for their participation. The study was approved by the ethics committee of the medical faculty of the University of Kiel, Germany and followed the ethical standards of the Helsinki Declaration.

### Participants

ADHD patients were referred to our study from the Department of Child and Adolescent Psychiatry of the University of Kiel. Healthy children were recruited through announcements in local newspapers, and healthy adults (all students) were recruited by announcements at the university. Since ADHD is diagnosed more often in boys than in girls [34] and emotional reactions can be biased by gender even in childhood [35], only male participants were included in the study.

All children and their parents were interviewed using a German translation of the Revised Schedule for Affective Disorders and Schizophrenia for School-Age Children: Present and Lifetime Version (K-SADS-PL) [36], [37]. A standard parent-reported questionnaire, the Child Behavior Checklist (CBCL) [38], was filled out by parents to assess any psychiatric symptoms of their children. Adults were screened for psychiatric symptoms by using the German version of the Structured Clinical Interview for DSM-IV (SKID-I/II) [39] and a German translation of a short version of the Symptom Check List (SCL-90-R) [40]. To ensure that adults did not exhibit any ADHD symptoms, they were asked to fill out the German short version of the Wender Utah Rating Scale (WURS-k) [41], in order to retrospectively assess any ADHD symptoms during childhood (cut off: >30), and the German self-rating behavior questionnaire ADHS-Selbstbeurteilungsskala (ADHS-SB) [41] to assess current ADHD symptoms (cut off: >15).

ADHD patients were excluded, if they displayed any comorbidity apart from oppositional defiant disorder (ODD). Controls were excluded, if they displayed any psychiatric abnormalities. Further exclusion criteria for all participants were below-average intelligence quotient (IQ < 85) as measured by the Culture Fair Intelligence Test 20-Revised Version (CFT 20-R) [42], significant memory impairment as measured by the Diagnosticum für Cerebralschädigung (DCS, cut-off score: <16th percentile of the reference sample) [43]. Adult participants were excluded, if they reported sleep-related disorders as measured with the German translation of the Pittsburgh Sleep Quality Index (PSQI, cut off >5) [44]. Children were screened for sleep-related disorders with a self-constructed questionnaire based on the PSQI which was conducted for clinical purposes and already used in a previous study [19]. Here, parents were asked to rate whether or not their children displayed symptoms of sleep disorders (e.g., sleep-related breathing disorders, frequent nocturnal awakenings, excessive daytime sleepiness; rating from never = 0 to always = 4). Parental rating data, as well as children's polysomnography (PSG) data, were evaluated by a somnological expert. Children were excluded if parental ratings in association with the PSG data showed any possibility of a sleep disorder.

Prior to the experiment, eight children were excluded from the ADHD group because they did not meet the DSM IV–TR [1] criteria for ADHD; three children were excluded from the control group because they displayed subclinical ADHD symptoms. Three children (two ADHD) quit the study during experimental sessions. Due to technical problems (no PSG recordings, corrupt behavioral data), the data of five participants (1x ADHD, 3x healthy children, 1x healthy adult) had to be excluded from the analyses.

The data of 16 children suffering from ADHD (aged 9–12), 16 healthy children (aged 9–12), and 20 adults (aged 20–28) were entered into analyses. Children with and without ADHD did not differ in age (t_30_ = .93, p = .361), and the groups did not differ with respect to IQ (F_2,49_ = 2.62, p = .083, see Table 1). All participants had normal or corrected-to-normal vision. Patients met the criteria for ADHD according to DSM IV–TR [1]; eight suffered from the inattentive type and another eight from the combined type. Four patients with ADHD additionally exhibited an oppositional defiant disorder (ODD). Healthy children and adults did not display any psychiatric abnormalities. In particular, none of the adults reported present or past ADHD symptoms (WURS-k sum score: M = 7.2; Range: 0–16; ADHS-SB sum score: M = 4.9; range: 1–10). According to self-reports all participants were free of any neurological, immunological, or endocrinological disease. No participant took any medication except twelve ADHD patients taking methylphenidate who discontinued medication 48 hours (approximately twelve half-lives) prior to each experimental condition. According to self-ratings (PSQI-Range: 0–5), parental ratings, and PSG data, all participants were free of sleep disorders. Compared to healthy children, parents of children suffering from ADHD reported restless sleep in their children more often (ADHD: M = 2.2; SD = 1.1; controls: M = 1.2, SD = .5; p = .004) as well as prolonged sleep latency (ADHD: M = 45.0 min, SD = 21.2; controls: M = 26.6 min, SD = 13.1; p = .025; comparison of all other scales were not significant, p>.05; data not shown). PSG data of ADHD patients did not confirm prolonged sleep latency (see Table 1) or restless sleep (no differences between children with or without ADHD with respect to sleep-efficiency, sleep stage change index, number of awakenings, or duration of wakefulness during the night of sleep).

**Table 1:** Participant Characteristics and Sleep Parameters.

	ADHD	Healthy Children	Healthy Adults		
	Mean (SD)	Mean (SD)	Mean (SD)	*F*	*p*
Age	10.6 (.95)^#^	11.1 (.95)^#^	24.7 (2.8)	334.9	**<.001**
IQ	105 (13.6)^#^	109 (10.6)^#^	114 (11.7)	2.7	.076
Figural memory	74.6 (19.4)	71.5 (23.7)	82.0 (19.1)	1.3	.285
TIB min	612 (57.4)^##^	574 (61.4)^##^	475 (51.2)	29.1	**<.001**
TST min	528 (57.2)^##^	507 (61.3)^##^	420 (55.4)	18.3	**<.001**
Number of awakenings	18.0 (6.1)	18.4 (7.6)	19.6 (7.6)	.22	.803
Wake after sleep onset (in min)	7.1 (6.7)	4.1 (3.0)	5.8 (5.0)	1.3	.275
Sleep efficiency %	86.6 (8.5)	88.3 (6.9)	88.5 (5.9)	.36	.699
Sleep onset latency	28.9 (28.3)	25.5 (17.8)	26.9 (14.6)	.13	.894
Sleep stages (in min)					
S1	30.0 (16.6)	25.4 (9.2)	32.5 (11.2)	1.5	.223
S2	224.7 (40.0)^#^	243.6 (68.6)^#^	211.6 (48.7)	1.7	.188
SWS	166.4 (26.3)^##^	155.2 (30.1)^##^	87.8 (30.9)	39.3	**<.001**
REM	107 (28.8)	103.2 (27.5)	90.9 (23.0)	1.9	.150
Sleep stage change index	18.0 (3.4)	17.5 (4,3)	19.5 (6.6)	.74	481
Oscillations (in μV^2^)					
SO during SWS	126.2 (49.8)^##^	132.4 (48.1)^##^	40.5 (22.8)	29.2	**<.001**
Delta during SWS	34.8 (13.0)^##^	38.8 (16.2)^##^	9.3 (5.3)	32.8	**<.001**
Sigma during S2	.28 (.20)^ ##^	.25 (.24)^ #^	.10 (.06)	5.6	**.006**
Theta during REM	.59 (.18)^##^	.64 (.24)^##^	.20 (.07)	35.1	**<.001**

Note: SD, standard deviation; TIB, time in bed; TST, total sleep time; sleep efficiency: ratio of total sleep time to time in bed, sleep onset latency: time in minutes from lights off to the first epoch of stage 2 sleep; S1, sleep stage 1; SWS, slow wave sleep; REM, rapid eye movement; SO, slow oscillation; bold p-values indicate significant main effect for groups; # (p≤.05) ## (p≤.005), significant difference in means compared to healthy adults; means did not differ between children with and without ADHD (p>.05).

### Emotional Memory Task

**Figure 1:**
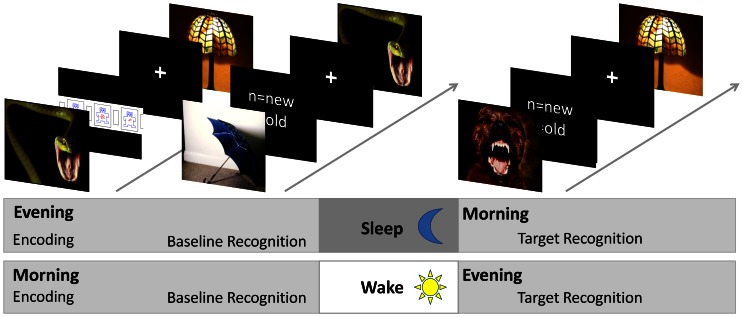
Design and picture recognition paradigm. During encoding, participants were shown 140 pictures and instructed to indicate how emotional they perceive each picture with respect to arousal using the computer-based version of the Self-Assessment Manikin (SAM) rating system [46]. Recognition memory was tested in an immediate recognition test, as well as in a delayed recognition test, after retention intervals with or without sleep. Picture recognition memory was assessed by presenting pictures from the encoding session (referred to as ‘old’ pictures) which were randomly mixed with the same number of new pictures. 40 old pictures were selected from the learning set in order to test baseline recognition memory, and the remaining 100 pictures were presented in the delayed (target) recognition test. After presentation of each picture, participants were to indicate whether they had seen the picture before (‘old’) or not (‘new’) by pressing a key.

In total, 560 pictures (50% emotionally negative and 50% neutral) were used. Pictures were divided into two sets. Each set consisted of 50% emotional and 50% neutral pictures. About 36% of all pictures (45% emotional and 26% neutral ones) were taken from the International Affective Picture System (IAPS) [45]. Since the IAPS contains several emotional pictures that are not suitable for children (e.g. dead bodies, open wounds) and distinctly neutral pictures were underrepresented for our purpose, we completed the stimulus sets by adding pictures (64%) from our own data base and matched with IAPS picture content (for validations see next paragraph). In the learning sessions, participants were asked to rate their emotional arousal evoked by 70 emotional and 70 neutral pictures. Each trial started with the appearance of a fixation cross (500 ms) followed by the target picture (1500 ms). Then, the Self Assessment Manikin Scale for Arousal (SAM) [46] appeared on the monitor and participants were supposed to judge their degree of emotional arousal while processing the picture by pressing one of nine response buttons (ranging from 1 = very low to 9 = very high). After the response was given, the next trial started. Immediately after encoding, a first retrieval session was carried out to achieve a recognition baseline. The introduction of a baseline session was necessary since children with ADHD not only display deficits in sustained attention in general [47] but also specific deficits in the encoding of visual stimulus material for later memory formation [48], [49]. In order to control for each individual level of encoding, 40 old and 40 new pictures were used, and participants were asked to rate whether or not they recognized pictures from the encoding session. Each trial started with the fixation cross (500 ms) followed by a target picture (1500 ms). Participants were asked to indicate whether or not they recognized pictures from the current encoding session (“old”) or not (“new”) by pressing one of two response buttons. The next trial did not start until an answer was given. The target recognition session took place after the retention interval of either sleep or wakefulness. Here, the remaining 100 old pictures together with 100 new pictures were presented. Again, participants were asked to rate whether or not they recognized pictures from the learning session. The trial timing was identical with baseline sessions (see also Figure 1). Stimulus presentation and response recording were conducted with E-Prime 2.0 (Psychology Software Tools, Inc., Pittsburgh, PA; http://www.pstnet.com).

Prior to the experiment, fourteen healthy adults (6 men) rated pictures of our own database by using the self-assessment manikin (SAM) scales for valence and arousal [45]. Comparison of picture ratings of our own pictures with IAPS normative ratings by t-tests revealed that valence rating did not differ between IAPS and our own pictures, neither in emotional (IAPS: M = 3.3, own data base: M = 3.4, p = .7) nor in neutral condition (IAPS: M = 5.1, own base: M = 5.3, p = .2). Our own pictures, however, were rated as more arousing compared to IAPS pictures (emotional pictures: IAPS: M = 5.3, own base: M = 6.5, p<.001; neutral pictures: IAPS M = 3.8, own base: M = 4.3, p<.001). Most importantly, IAPS and our own emotional pictures were strikingly rated as being less pleasant and more arousing compared to neutral ones (combined: p<.001; IAPS: p<.001; own pictures p<.001).

### Procedure

Each participant underwent testing in two conditions. In the sleep condition, learning and baseline measurements were carried out in the evening right before sleep, while target recognition took place after a night of sleep. In the wake condition, learning and baseline measurements were conducted in the morning, and target recognition was held in the evening of the same day. Both conditions were conducted in participants' home environment to avoid any inconveniences caused by sleep laboratory condition. We also adapted times of learning and recognition in order to uphold the participants' daily time schedule and avoid shifts in the sleep-wake behaviour (for children the morning session was at 08:15 a.m. and the evening session at 07:30 p.m.; for adults the morning session was at 07:30 a.m. and the evening session at 09:30 p.m.). To control for possible effects of session or picture sets, the order of conditions (each being conducted at least two weeks apart) and picture sets was counterbalanced within each group.

At the beginning of each session, participants were requested to rate their current emotional state using the SAM scales for valence, arousal, and dominance. To assess current level of alertness, participants had to complete the subtest for alertness of the Testbatterie zur Aufmerksamkeitsprüfung für Kinder (KITAP) [50]. In this computer-based Go-paradigm participants were asked to pay attention to a computer screen and every time a certain visual stimulus occurred, they had to press a response button as fast as possible.

The sleep condition consisted of two consecutive nights. The first night was used for adaptation: Participants familiarized themselves with the polysomnographic (PSG) recording system by sleeping with a dummy recording system. The following day, the sleep condition continued, including behavioral and PSG recordings. Physiological signals were recorded using an 8-channel Somnomedics PSG system (Randersacker, Germany) to record electroencephalogram (EEG), electrooculogram (EOG), and submental electromyogram (EMG). Bipolar EEG was recorded at a 128-Hz sampling rate with band-pass filter (0.2–75 Hz) according to the International 10–20 system from F3, F4, C3, C4, A1, and A2 referenced to Cz with a ground placed at AFz. Diagonal EOG (sampling rate: 128 Hz, band-pass filter: 0.2–75 Hz) was recorded from the lower right and higher left canthi. EMG was recorded at 256-Hz with a band-pass filter set to 0.2-128 Hz.

In the experimental nights the following was measured: time in bed (TIB), sleep onset latency (time in minutes from lights off to the first epoch of sleep stage 2), total sleep time (in minutes), sleep efficiency (ratio of total sleep time to time in bed), number of awakenings, duration of wakefulness after sleep onset, sleep stages 1–4 and REM sleep (in minutes), and sleep stage change index (number of sleep stage changes per hour of sleep). Sleep stages were visually scored according to standard criteria (C3-A2, C4-A1) [51] by a trained rater. Oscillatory EEG activity was obtained from frontal electrodes. Because electrodes over F3 lost skin contact during nocturnal recordings in five participants (2x ADHD, 3x healthy controls) but only in one participants over F4 (1x ADHD), we relayed power analyses on F4, which was referenced against the mean of A1 and A2. The fast Fourier transform (FFT) algorithm was performed using Brain Vision Analyzer 2 (Brain Products, Germany). Only artefact-free epochs of 10-second duration were analysed, and the truncating error was reduced by a Hanning window. The log-transformed absolute power values for low delta (0.6–1 Hz) and delta (1–4 Hz) during SWS (S3+S4), sigma (11–16 Hz) during S2, and theta (4–7 Hz) during REM sleep were used for further analyses.

During picture encoding, the electrodermal activity (EDA) was recorded with a frequency range of 0–10 Hz using two Ag/AgCl electrodes (4 mm inner diameter) placed on the thenar and hypothenar of the non-dominant hand; the ground was placed at the forehead. The EDA data were recorded, amplified, and filtered with V-Amp (Brain Products, Germany) and sampled at 500 Hz. The raw EDA signal was low-pass filtered offline (0.64 Hz, 24 dB/octave) [52] and then corrected for baseline (500-0 ms before stimulus onset). The skin conductance response (SCR) was extracted as the maximum deflection in an interval from 1000 to 4000 ms after stimulus onset [53]. Trials were visually inspected for data quality, and trials with excessive deflection (peak exceeding 3SD individual mean reaction) were excluded. Due to technical problems, four participants (one ADHD child and three adults) did not contribute data to the analysis at all. In eleven ADHD children, 12 healthy children, and seven adults, electrodes lost skin contact either in the sleep or the wake condition. Therefore, SCR data of both sleep and wake condition were combined. To eliminate individual differences in responsivity and permit a meaningful summation of the responses of different participants, single SCR were standardized by z-transformation within each participant [54]. Within-series z-scores were selected, because they are more resistant to habituation effects [55]. Finally, mean standardised SCRs were calculated for emotional and neutral conditions within each participant.

### Statistical Analyses

Recognition accuracy was computed by subtracting false-alarm rates from hit rates [56]. For sleep and wake conditions, target recognition accuracy was subtracted from baseline recognition accuracy (baseline-target). Data from this baseline-corrected recognition accuracy were included in a repeated measurement analysis of variances (ANOVA). The ANOVA contained a between-subject factor GROUP (ADHD vs. healthy children vs. healthy adults), a within factor SLEEP (sleep vs. wake condition) and a within factor EMOTION (emotional vs. neutral pictures). Sleep-associated changes in emotional bias (i.e. difference between recognition accuracy of emotional and neutral pictures) are indicated by an interaction between the factors SLEEP×EMOTION; predicted differences in groups were expected to result in a significant interaction SLEEP×EMOTION×GROUP. In order to test group differences in sleep-dependent emotional memory bias a priori t-contrasts were used.

Polysomnographic data were compared between groups by employing one-way ANOVAs. Correlations between sleep parameters and sleep-dependent emotional bias were performed by Pearson's correlation coefficients. Fisher's z-test (two-tailed) was used for group comparison of correlation coefficients.

Emotional picture rating was analyzed by using an ANOVA with the factors GROUP, SLEEP, and EMOTION. SCR data were analyzed by using an ANOVA with the within factor EMOTION and the between factor GROUP. Emotional self ratings (SAM scales for valence, arousal and dominance) and KITAP alertness scores were analyzed by separate ANOVAs, each employing the between factor GROUP and the within factors SLEEP and DAYTIME (morning vs. evening). Post-hoc comparisons of single means were analyzed using Student's t-tests. All tests were performed two-tailed, a p-value of <.05 was considered statistically significant.

## Results

### Emotional Reactions Evoked by Pictures

The analyses of picture ratings during learning sessions revealed a main effect for EMOTION (F_1,49_ = 92.2, p<.001), which reflects the fact that emotional pictures were rated as being strikingly more arousing than neutral ones (emotional: M = 5.0, SEM = .21; neutral: M = 2.6, SEM = .17). Neither the interaction between EMOTION and GROUP (F_2,49_ = 1.57, p = .217) nor the main effect for GROUP (F_2,49_ = .59, p = .556) was significant. Explorative post-hoc tests revealed that all groups rated emotional pictures as more arousing (ADHD: t_15_ = 4.28; p = .001; healthy children: t_15_ = 6.67, p<.001; healthy adults: t_19_ = 4.09, p = .001). While there was a marginal difference in the rating of neutral pictures between children with and without ADHD (ADHD: M = 3.1, SEM = .1.6; healthy children: M = 2.2; SEM = .9; ADHD vs. healthy children: t_30_ = 2.04, p = .050) all other comparisons of single means between groups were not significant (p>.186).

As shown by the main effect for EMOTION (F_1,45_ = 5.03, p = .030), emotional pictures evoked a higher standardized SCR than neutral pictures did (emotional: M = .028, SEM = .012; neutral: M = −.028, SEM = .012). The interaction between GROUP and EMOTION was not significant (F_2,45_ = 2.08, p = .136). Due to z-transformation, SCR values within each participant (and within each group) summed up to zero; therefore, a main effect for GROUP could not be obtained.

### Emotional Memory Task

**Figure 2:**
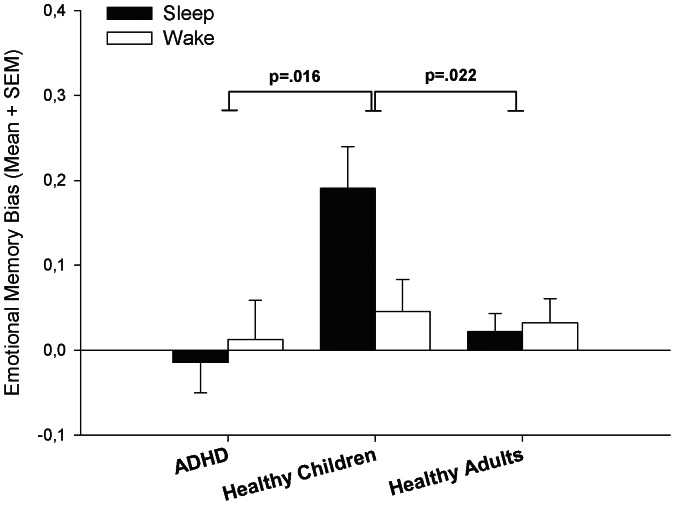
Behavioural performance; emotional memory bias, difference in recognition accuracy between emotional and neutral picture condition; note that all values are baseline-corrected and indicate changes in emotional bias over retention intervals; SEM, standard error of means.

There were main effects for all three factors EMOTION, SLEEP, and GROUP which indicated that, in general, emotional pictures were better recognized than neutral ones (F_1,49_ = 9.14, p = .004), sleep enhanced the picture recognition accuracy (F_1,49_ = 33.71, p<.001) and that groups differed in performance (F_2,49_ = 3.56, p = .036). The interaction EMOTION×GROUP (F_2,49_ = 4.81, p = .012), and, as predicted, the interaction SLEEP, EMOTION and GROUP (F_2,49_ = 3.86, p = .028) reached significance. A priori t-contrasts revealed that sleep-associated emotional bias was higher in healthy children compared to ADHD patients (t_49_ = 2.5, p = .016) and to healthy adults (t_49_ = 2.36, p = .022), but did not differ between ADHD patients and healthy adults (t_49_ = .29, p = .804). The corresponding descriptives are shown in Table 2 and Figure 2. In order to test for possible effects of individual emotional engagement during encoding, we used absolute differences in ratings between emotional and neutral pictures as a covariate and reanalyzed behavioural data employing an analysis of covariance (ANCOVA). However, the interaction between SLEEP, EMOTION, and GROUP was still significant (F_2,49_ = 3.41, p = .041). Moreover, an additional analysis of behavioural data was performed with respect to individual emotional ratings during encoding. Here, within each participant pictures were divided post-hoc into high-arousal and low-arousal pictures based on median split of arousal ratings during encoding. However, behavioural data (i.e. hit rates) in terms of post-hoc and a priori defined emotional categories did not differ within any group (ADHD: p = .418; healthy children: p = .245; healthy adults: p = .566).

**Table 2:** Behavioural Data.

			ADHD	Healthy Children	Healthy Adults
			Mean (SEM)	Mean (SEM)	Mean (SEM)
Sleep	Baseline	Emotional	.82 (.04)	.80 (.03)	.86 (.01)
		Neutral	.69 (.03)	.89 (.02)	.86 (.02)
	Target	Emotional	.62 (.04)	.79 (.02)	.70 (.02)
		Neutral	.50 (.04)	.68 (.03)	.69 (.02)
	Target-Baseline	Emotional	−.20 (.04)	−.02 (.03)	−.16 (.02)
		Neutral	−.19 (.03)	−.21 (.04)	−.18 (.02)
	Target-Baseline	Emotional Bias	−.01 (.04)	.19 (.05)	.02 (.02)
Wake	Baseline	Emotional	.78 (.04)	.85 (.02)	.87 (.02)
		Neutral	.63 (.05)	.80 (.04)	.84 (.02)
	Target	Emotional	.50 (.04)	.64 (.03)	.59 (.03)
		Neutral	.34 (.05)	.54 (.03)	.54 (.03)
	Target-Baseline	Emotional	−.28 (.03)	−.21 (.03)	−.27 (.03)
		Neutral	−.29 (.03)	−.25 (.03)	−.31 (.03)
	Target-Baseline	Emotional Bias	.01 (.05)	.05 (.04)	.03 (.03)
Sleep-Wake	Target-Baseline	Emotional Bias	−.03 (.05)	.14 (.06)	−.01 (.03)

Note: Displayed values refer to recognition accuracy, SEM, standard error of means.

### Polysomnographic Data

As revealed by one-way ANOVAs and subsequent t-tests, healthy children, as well as children with ADHD, displayed prolonged TIB, TST, S2, and SWS (see also Table 1) compared to healthy adults. Similarly, children with and without ADHD showed higher SO/delta power during SWS, higher sigma power during S2, and higher theta power during REM sleep. Sleep parameters did not differ between children with and without ADHD (p>.2, except TIB: p = .086).

### Correlation Analyses

**Figure 3:**
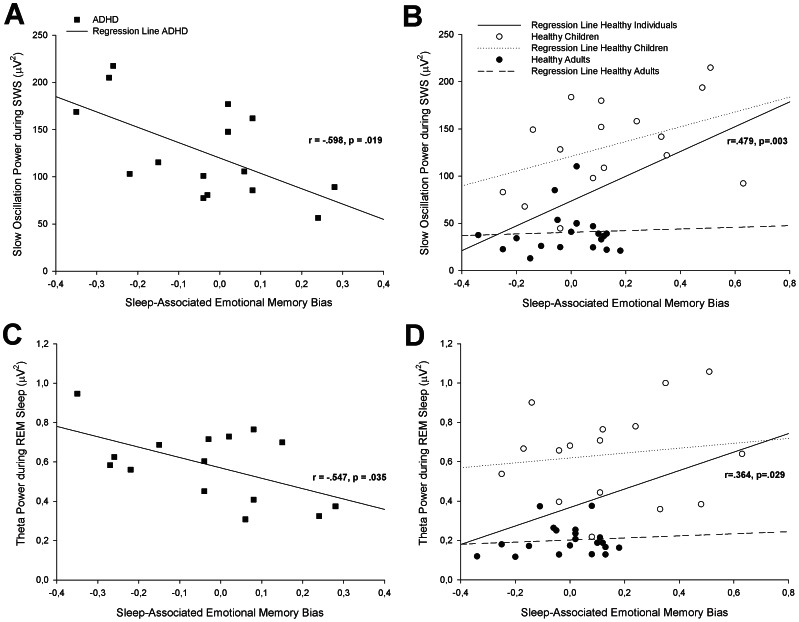
Correlations between sleep-associated emotional memory bias and slow oscillation power during SWS (A in ADHD and B in healthy children/adults) and theta power during REM sleep (C in ADHD and D in healthy children/adults), SWS, slow wave sleep, REM, rapid eye movement.

In order to test the hypothesis that oscillatory activity during sleep supports memory consolidation in healthy individuals, we first calculated correlation coefficients separately for healthy children and healthy adults. As presented in Table 3, correlation coefficients did not reach significance in healthy children or in healthy adults. However, since we expected the same memory-supporting mechanism of oscillations during sleep in healthy children and in healthy adults (note that correlation coefficients between healthy children and healthy adults did not differ; p>.284), we merged the data from healthy children and adults. After combining data from healthy individuals, we found positive correlations between sleep-associated emotional memory bias and SO power during SWS in healthy individuals (r = .479, p = .003, see Table 3 and Figure 3). In contrast, children with ADHD displayed a negative correlation between performance and SO power during SWS (r = −.598, p = .019), which also differed from that in healthy individuals (z = 3.59, p<.001). The same was true for delta power during SWS (ADHD: r = −.547, p = .035; healthy individuals: r = .423, p = .010; ADHD vs. healthy individuals: z = 3.16, p = .002). Likewise, theta oscillations during REM sleep correlated negatively with memory performance in children with ADHD (r = −.547, p = .035) but positively in healthy individuals (r = .364, p = .029; ADHD vs. healthy individuals: z = 2.95, p = .003). There was no association between emotional memory performance and sigma power during S2 (p>.198), and correlation coefficients did not differ between groups (p>.166).

**Table 3:** Correlations between sleep parameters and sleep-associated emotional memory bias.

	ADHD		Healthy Children		Healthy Adults		Healthy Individuals	
	r	*p*	r	*p*	r	*p*	r	*p*
SWS (min)	−.270	.312	−.286	.283	.259	.271	.244	.152
SO	**−.598**	**.019**	.421	.104	.054	.822	**.479**	**.003**
Delta	**−.547**	**.035**	.282	.289	.035	.885	**.423**	**.010**
REM (min)	−.095	.726	.318	.231	−.208	.380	.200	.242
Theta (REM)	**−.547**	**.035**	.135	.619	.100	.675	**.364**	**.029**
S2 (min)	.021	.938	−.328	.216	−.347	.134	−.193	.260
Sigma	.228	.415	.006	.983	−.301	.198	.124	.472

Note: HI, healthy individuals; SWS, slow wave sleep; SO, slow oscillations; REM, rapid eye movement; bold values indicate significant correlation coefficients.

### Emotional Self Ratings

In the beginning of each session, participants were asked to rate their emotional state on the SAM scales “valence”, “arousal”, and “dominance” [45]. The analysis of emotional self-ratings revealed a main effect for DAYTIME with respect to the SAM scale “valence” (F_1,49_ = 5 .56, p<.022), indicating that all participants were in a better mood in the evening than in the morning session (evening: M = 2.21, SEM = .15, morning M = 1.92, SEM = .20). All further main effects or interactions effects did not reached significance (p>.064). Particularly, the interactions between SLEEP×DAYTIME×GROUP within all three SAM scales were not significant (p>.336)

### Alertness

To test participants acute alertness, in the beginning of each session, participants performed the KITAP [50] subtest for alertness. The main effect GROUP (F_1,49_ = 23.9, p<.001) revealed that, in general, ADHD patients displayed the prolonged reaction times (M = 328.2) compared to healthy children (M = 284.0; t_30_ = 2.39, p = .022) and healthy adults (M = 231.8; t_34_ = 6.25, p<.001); healthy adults showed shortest RT (healthy children vs. healthy adults: t_34_ = 6.84, p<.001). Other main effects and interactions between the factors SLEEP, DAYTIME, and GROUP were not significant (p>.191).

Sleep-associated emotional memory bias was not correlated with IQ (p = .8), age (p = .3), emotional picture rating (p = .3), or duration of retention interval (p = .9).

## Discussion

Here, we investigated the sleep-dependent consolidation of emotional memory in children with and without ADHD in the context of healthy development. As presumed, children with ADHD displayed deficits in sleep-associated consolidation of emotional memory which correlated negatively with frontal EEG activity during sleep. Moreover, we found a decline in sleep-associated consolidation of emotional memory from healthy children to healthy adults. Before discussing deviant memory processes in children with ADHD, we first discuss effects of age on sleep-related memory performance in healthy children and adults.

While several studies reported a benefit from sleep with respect to emotional memory in healthy individuals [17]–[19], [25], [57]–[59], our results showed for the first time that healthy children outperform healthy adults. As memory performance was baseline-corrected, the results revealed that the healthy children displayed a diminished emotional memory bias only in the wake but not in the sleep condition. Sleep, however, had no impact on the loss of the emotional memory in healthy adults which is in contrast to most other published studies (but see also[60], [61]). In fact, emotional stimulus ratings and skin conductance reactions indicated that emotional pictures were strikingly more arousing and that emotional reactions did not differ between groups. We cannot rule out, however, that the lack of sleep-related emotional bias in healthy adults might be caused by rather “weak” (i.e. child-oriented) stimulus material. At the same time, we found correlations between memory performance and oscillatory activity during sleep only when combining healthy children with healthy adults. This procedure assumes that oscillatory activity during sleep supports memory consolidation in healthy children and adults equally. As yet, there exists no compelling evidence. However, there are studies that show that SWS, as well as slow oscillatory activity during SWS, in healthy children coincide with hippocampus-dependent memory performance, as has been shown before in adults [27]. In accordance with these reports, the correlation coefficients did not differ between healthy children and adults in our sample. When combining data of healthy individuals, emotional memory bias correlated positively with frontal slow and delta oscillation power during SWS as well as with theta power during REM. According to the system consolidation hypothesis, new memory representations are reactivated during SWS, as well as during REM sleep, in order to be integrated into already existing memory systems. Here, the PFC may play a critical role: frontally generated slow oscillations during SWS are believed to drive the reactivation of hippocampus-associated memory representation [62], while frontal theta power during REM sleep might reflect the memory boosting synchronisation of PFC and amygdala activity during REM sleep [23]. Evidence from human fMRI and animal studies suggests that the sleep-dependent consolidation of emotional memory is related to an integration of both amygdala and hippocampus activity [20], [24]. Therefore, we assume that SO activity during SWS (emphasizing the hippocampus-dependent aspects of emotional memory) and theta-activity during REM sleep (emphasizing amygdala-dependent aspects of emotional memory) reflect two parts of a sequential process in emotional memory consolidation during sleep. A similar sequential role of sleep was proposed in animals and humans for several memory systems including avoidance learning [63], [64]. In accordance with others [26], [28], we found that SWS and slow wave activity (1–4 Hz) was increased in healthy children compared to adults; the same was true for theta power during REM sleep.

Taking this further, our data indicate that the selective function of sleep in contrasting emotional from non-emotional memory representations is linked to pronounced EEG activity during SWS and REM sleep. The diminished emotional memory performance in adults, however, might provide critical conditions of sleep-associated consolidation of emotional memory: sufficiently oscillatory EEG activity during sleep in combination with sufficiently arousing material is required to substantially improve emotional memory performance after sleep. These interpretations, however, should be taken with caution since correlation coefficients only showed significance after data of both healthy groups were combined.

As predicted, children with ADHD compared to healthy children displayed less of a sleep-dependent emotional bias. However, emotional ratings during encoding indicate that children with ADHD rated neutral pictures as being slightly more arousing than healthy children did. This could bring up the concern that differences in memory performance between children with and without ADHD are caused more by insufficient differentiation between emotional and neutral stimuli during encoding than by deviant emotional memory consolidation during sleep. However, it seems unlikely that the complete lack of sleep-associated consolidation of relevant memory within the ADHD group is based on relatively small between-group differences in ratings, since a) emotional pictures in children with ADHD were rated as significantly more arousing than neutral ones (p = .001), b) reanalysis of behavioural data based on emotional ratings (ANCOVA, median split) did not affect this outcome and c) the correlation analyses indicated that differences in performance between children with and without ADHD is associated with sleep EEG-patterns than with rating data during encoding. Therefore, we assume that in ADHD the lack of contrasting between relevant and irrelevant memory representations by sleep can be ascribed to an aberrant function of frontal oscillatory EEG-activity during SWS and REM sleep.

Although parents of patients reported prolonged sleep latency and restless sleep in their children, we could not confirm these ratings and did not find differences in sleep parameters between children with and without ADHD. With respect to the results of a recent meta-analysis which showed that sleep problems in children with ADHD are judged to be more pronounced in parental reports than in objective sleep studies [14], it cannot be ruled out that possible sleep problems (e.g. higher bed time resistance, difficulties with morning awakenings) in children with ADHD were covered by our strict experimental protocol. While we and others did not find differences between ADHD and healthy controls in oscillatory activity during sleep [16], [65], a recent study using high-density EEG revealed altered anterior-posterior distribution of slow wave power in ADHD [33]. Without manipulating oscillatory activity directly, however, it remains unclear whether altered oscillatory activity itself caused memory consolidation in ADHD or rather reflected dysfunctional interplay between memory-related brain regions. Several studies provided evidence for structural and functional alterations in the PFC, the hippocampus, the amygdala, and the interplay between them [5], [7], [8], [66], [67]. Besides the important role of the amygdala and the hippocampus in emotional memory bias [9]–[11], the PFC is assumed to tag those representations which are detected as future-relevant during encoding [68] in order to organize their reactivations preferentially during subsequent sleep [27]. As children with ADHD not only display deficits in sustained attention in general [47] but also specific deficits in the encoding of visual stimulus material for later memory formation [48], [49], a baseline condition was introduced. Hereby, memory performance in the retrieval sessions is susceptible to different levels of encoding; however, we want to point out that in a previous study without controlling for effects of encoding we observed a similar but attenuated impact of sleep on emotional memory bias in healthy children and children with ADHD [16]. Since behavioural data were baseline-corrected, the results stress the role of sleep in aberrant emotional memory performance in ADHD. However, the exact pattern of the underlying deviant brain activity during sleep can only be investigated in further imaging studies.

From a clinical standpoint, the lacking contrast between emotional and neutral memory representations after sleep in ADHD could give insight into aberrant development of emotional function in ADHD. Due to a lack of reorganization of emotional content in children with ADHD, one can assume that emotional problems during the daytime are amplified by dysfunctional sleep. Indeed, ADHD is often accompanied by deficits in emotional processing [2]–[4], and comorbidities of emotional disorders are common in ADHD [69], [70]. Epidemiological reports revealed that patients with ADHD are more likely to display risky behaviour and are less affected in their behaviour by negative experiences [71], [72]. Considering that the function of sleep in emotional memory consolidation seems to be pronounced among healthy youth, further studies in adult patients suffering from ADHD are required to clarify whether the selective function of sleep is regained after puberty or whether our results reflect a fundamental dysfunction which is independent of a maturational delay [66].

Taken together, we propose that frontal SO power during SWS in combination with theta activity during REM sleep in healthy individuals has the function to shape the contrast between relevant (emotional) and irrelevant (neutral) memory representations. Results obtained in children suffering from ADHD confirm the hypothesis that frontal brain activity during sleep is critically involved in this function of sleep.
